# Supplementary material for: The Impact of Preconception Gastric Bypass Surgery on Maternal Micronutrient Status before and during Pregnancy: A Retrospective Cohort Study in the Netherlands between 2009 and 2019
Source: Nutrients. 2022 Feb 9;14(4):736. doi: 10.3390/nu14040736 (PMC8876006; doi:10.3390/nu14040736)
Supplement: Supplementary file 1 [file nutrients-14-00736-s001.zip › nutrients-1541267-supplementary.pdf]

Table S1. Intraclass correlation coefficients within pregnancy, within persons and residuals for hemoglobin and micronutrient status.

	Hemoglobin		Folate		Calcium		Iron		Vitamin B12		Vitamin D		Ferritin	
**Within pregnancy**	0.097	24%	39.9	31%	0.00113	13%	5.91	15%	0.0572	15%	80.3	22%	972	25%
**Within person**	0.121	30%	17	13%	0.00193	23%	3.7	9%	0.0271	7%	70.1	19%	495	13%
**Residual**	0.184	46%	70.9	55%	0.00551	64%	29.8	76%	0.297	78%	214	59%	2438	62%

Table S2. Quantities of supplements compared to the reference intake.

	**FFM®** Quantity (% of reference intake pregnant women)	**Davitamin Compleet Mama** Quantity (% of reference intake pregnant women)	**Reference intake** Quantity
**Iron**	70 mg (350%)	16.1 mg (80.5%)	20 mg
**Folic acid**	600 µg (150%)	400 µg (100%)	400 µg
**Vitamin B12**	350 µg (13462%)	2.5 µg (96.2%)	2.6 µg
**Vitamin D**	75 µg (1500%)	10 µg (200%)	5 µg
**Calcium**	-	120 mg (10%)	1200 mg
